# A high-yielding recipe: Cytokinin signaling in soybean roots affects phosphorus uptake efficiency and crop production

**DOI:** 10.1093/plphys/kiad616

**Published:** 2023-11-17

**Authors:** Amy Lanctot

**Affiliations:** Assistant Features Editor, Plant Physiology, American Society of Plant Biologists; Cold Spring Harbor Laboratory, Cold Spring Harbor, NY, USA 11724

As autotrophs, plants require relatively little input to produce both vegetative and reproductive tissue but still need key nutrients for growth and development, particularly nitrogen, potassium, and phosphorus. These 3 elements are the major components of commercial fertilizers, though the efficacy of these fertilizers relies on the efficient uptake of these nutrients from the soil by plant root systems. Increasing nutrient uptake efficiency is consequently a key target in agricultural research. The less supplemental fertilizer, sourced from nonrenewable mineral deposits, is required by crops, the more economical, sustainable, and environmentally sensitive production of these crops can be (Fageria et al. 2008). Mineral nutrients are taken up from surrounding soil by roots, and plant root system architecture can change drastically in response to nutrient availability to seek out deficient nutrients. Much of this plasticity in root growth is regulated by hormones that affect the production of primary and lateral roots that shapes a root system's architecture (López-Bucio et al. 2003). Particularly the balance in the root meristem between cell division, promoted by the hormone auxin, and cell differentiation, promoted by the hormone cytokinin, can affect many physiological traits in root architecture to build a root system that responds in real time to nutrient availability (Chapman and Estelle 2009).

In this issue of *Plant Physiology*, Yang et al. use quantitative trail loci (QTL) and genome-wide association study (GWAS) mapping approaches to identify variation in a gene locus that affects soybean accessions’ ability to tolerate low-phosphorus conditions. The authors first mapped QTLs associated with soybean plant height in normal and low-phosphorus soils within a recombinant inbred line panel generated from a cross between a low-phosphorus–tolerant soybean variety and a low-phosphorus–sensitive soybean variety. They identified 14 QTLs that explained variation in low phosphorus tolerance between these accessions. They then performed a GWAS for plant height among 219 accessions of soybean in these same conditions. By finding overlap in their results from these 2 approaches, they found single nucleotide polymorphisms identified in the GWAS that lie in 1 of their QTLs. This locus encoded a gene homologous to *ARABIDOPSIS RESPONSE REGULATOR 1*, a cytokinin response transcription factor, which they called *GmRR1*. Characterizing *GmRR1* tissue-specific expression by quantitative PCR, they found that *GmRR1* is expressed most highly in the root, and its expression changes in response to low-phosphorus conditions, initially increasing and then decreasing.

Generating knockout mutants and overexpression lines of *GmRR1*, the authors found that these transgenic plants had substantially changed growth habits from wild type in both normal- and low-phosphorus conditions. Knockout mutants had expanded root systems (Fig. 1A), which caused increased plant height, root and shoot biomass, shoot branching, seed production, and phosphorus uptake efficiency (Fig. 1B), all agronomically important traits. These quantitative traits increased more substantially under low-phosphorus conditions, suggesting these mutants can better tolerate low-phosphorus soil. Conversely, overexpression lines showed reduced root systems (Fig. 1A), resulting in less biomass in both aerial and root tissue and affecting seed yield negatively as well (Fig. 1B). On the cellular level, knockout mutants had larger root meristems and increased root hair production, both developmental processes activated by auxin and repressed by cytokinin, suggesting that *GmRR1*, like its ortholog in Arabidopsis, promotes cytokinin signaling and antagonistically decreases auxin response. Interestingly, under phosphorus-deficient conditions, the knockout mutants show increases in both auxin and cytokinin levels. This suggests that inhibition of cytokinin signaling in these mutants occurs at the level of perception, which may cause feedback regulation that promotes cytokinin synthesis or transport (Fig. 1C).

**Figure 1.**
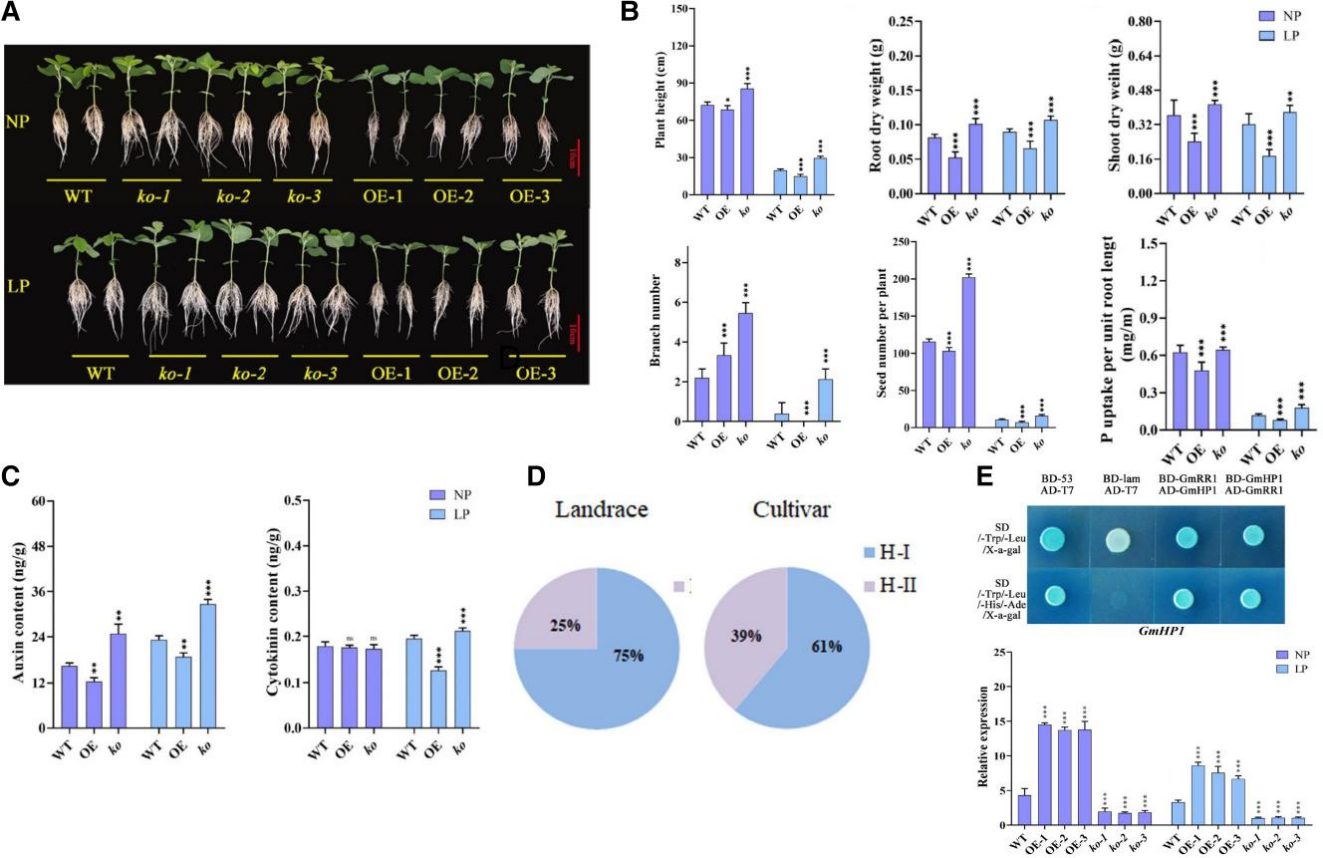
GmRR1 affects tolerance to low phosphorus in soybean by affecting cytokinin perception and root system architecture. **A)***GmRR1* knockout mutants show expanded root systems in soybean seedings compared to wild-type plants, whereas overexpression of *GmRR1* lines shows smaller root systems than wild type. **B)***GmRR1* knockout and overexpression affects plant height, root and shoot dry weight, shoot branching, seed production, and phosphorus uptake efficiency in both normal-phosphorus (NP) and low-phosphorus (LP) conditions. **C)** Auxin content is decreased in overexpression *GmRR1* lines and increased in *GmRR1* knockout lines in both NP and LP conditions. Cytokinin content is decreased in overexpression *GmRR1* lines and increased in *GmRR1* knockout lines but only in LP conditions. **D)** The *GmRR1* haplotype associated with LP tolerance is more common in domesticated soybean varieties than landrace accessions. **E)** Reciprocal yeast-2 hybrid shows the interaction between *GmRR1* and *GmHP1*. Quantitative PCR analysis shows *GmHP1* expression is affected in *GmRR1* overexpression and knockout lines in both NP and LP conditions.

To determine how *GmRR1*'s function in transcriptional regulation affects root development and phosphorus limitation response, the authors analyzed RNA-sequencing from roots of their transgenic lines in normal-and low-phosphorus conditions. They found that cytokinin synthesis genes were indeed increased in knockout mutants in low-phosphate conditions, as suggested by their hormone quantification data. Phosphate transporter genes were upregulated in these mutants as well, a possible mechanism by which uptake efficiency is increased. Auxin transport and auxin degradation genes were both decreased in the knockout mutants, as cytokinin signaling acts antagonistically to auxin response. The authors finally conducted an evolutionary analysis to determine whether the genomic region containing the *GmRR1* locus shows any signatures of selection. They found a strong selective signature on this locus, as the haplotype identified in their GWAS analysis as more prevalent in low-phosphorus tolerant accessions was much more frequently found in cultivated accessions compared to landraces (Fig. 1D) and in particular was nearly universal in soybean varieties from Brazil, a major soybean producer. This analysis suggests that *GmRR1* may have been a target during soybean domestication to promote soybean cultivation in phosphorus-deficient soils.

This research highlights the absolute essentiality of feedback regulation and hormone interactions during hormone signaling, as the activity of *GmRR1* is intrinsically linked to both interactions with other hormone signaling pathways as well as feedback on cytokinin signaling itself. *GmRR1* is a transcription factor that promotes cytokinin nuclear responses, some of which are counterintuitively negative regulation of cytokinin production and transport itself. Negative feedback relationships like this are common network motifs in plant hormone signaling pathways that allow for acute and rapid responses to environmental changes. The authors found that low-phosphorus conditions caused a transient increase in *GmRR1* expression followed by a decrease in its expression, suggesting such a negative feedback pathway on cytokinin may be a native soybean response to phosphorus limitation. They also found *GmRR1* function affects the signaling of auxin and ethylene, both hormones that change root system architecture and phosphorus uptake efficiency. Through these extensive interactions among hormone responses, complex and plastic responses in root elongation and branching can be built into root development to respond to nutrient availability.

Interestingly, the authors show that *GmRR1* both physically interacts with and affects the expression of the *GmHP1* (Fig. 1E), a histidine-containing phosphotransmitter that promotes cytokinin signal transduction. *GmHP1* is also downregulated in response to low-phosphorus stress, suggesting that low phosphorous can cause a substantial rewiring of hormone response globally in soybean. *GmRR1* knockout plants have substantially lowered expression of *GmHP1* in both normal- and low-phosphorus conditions, and *GmRR1* overexpression plants show increased expression of *GmHP1*. This result illustrates the extent to which transcriptional feedback regulation is embedded within cytokinin signaling, as loss of one signaling component strongly affects the expression of that component's interacting factors, further affecting the capacity for a strong signaling response. Exploring the circuitry of the hormone interaction and feedback relationships affected by *GmRR1* function is an area of potential research that would expand our capacity to build nutrient deficiency tolerance in this important crop species.

Isolating *GmRR1* as a key regulator of phosphorus deficiency response by using GWAS and QTL mapping approaches indicates that combining these 2 approaches can be a strategy used more broadly to identify loci that may contribute to desirable agricultural traits in many different crop species. Finding the loci that contribute to differences between environmentally robust and sensitive varieties, or between a high-yielding architecture and a low-yielding one, can inform engineering of these traits. For example, using these approaches, growers can modify elite accessions to environmentally robust haplotypes or instead grow natural varieties with haplotypes that are the most likely to succeed in their growth conditions. It is particularly interesting that the authors found strong selective signatures in *GmRR1* and that accessions that are grown in high-yield environments have been selected at this locus to be more tolerant to low-phosphorus conditions. Further work could expand this approach to other nutrient deficiencies. Particularly given the environmental impacts of nitrogen fertilizer on watersheds and algal blooms, increasing nitrogen uptake efficiency through such techniques could be particularly impactful (Dimkpa et al. 2020). As the world population increases, a growing concern is how to provide equitable access to nutritious food production. This work shows that a combination of natural population mapping and molecular phenotyping can identify strong candidate gene targets for precision engineering of the next generation of environmentally robust crops.

## Dive Curated Terms

The following phenotypic, genotypic, and functional terms are of significance to the work described in this paper:

cytokinin content Planteome: TO:0002660

mineral CHEBI: CHEBI:46662

phosphate CHEBI: CHEBI:18367

## References

[kiad616-B1] Chapman EJ , Estelle M. Cytokinin and auxin intersection in root meristems. Genome Biol. 2009:10(2):210. 10.1186/gb-2009-10-2-210

[kiad616-B2] Dimkpa CO , Fugice J, Singh U, Lewis TD. Development of fertilizers for enhanced nitrogen use efficiency—trends and perspectives. Sci Total Environ. 2020:731:139113. 10.1016/j.scitotenv.2020.139113 32438083

[kiad616-B3] Fageria NK , Baligar VC, Li YC. The role of nutrient efficient plants in improving crop yields in the twenty first century. J Plant Nutr. 2008:31(6):1121–1157. 10.1080/01904160802116068

[kiad616-B4] López-Bucio J , Cruz-Ramírez A, Herrera-Estrella L. The role of nutrient availability in regulating root architecture. Curr Opin Plant Biol. 2003:6(3):280–287. 10.1016/S1369-5266(03)00035-9 12753979

